# The characteristics of the chloroplast genome of the *Michelia chartacea* (Magnoliaceae)

**DOI:** 10.1080/23802359.2020.1871432

**Published:** 2021-02-11

**Authors:** Yanyan Li, Meng Zhou, LingMin Wang, Junqing Wang

**Affiliations:** Pingdingshan University, Pingdingshan, Henan, China

**Keywords:** *Michelia chartacea*, chloroplast genome, Magnoliaceae, phylogenetic analysis

## Abstract

In this study, the complete chloroplast genome of *Michelia chartacea* B. L. Chen & S. C. Yang was 160,138 bp in length. It includes a large single-copy (LSC) region of 88,164 bp, a small single-copy region (SSC) of 18,824 bp, and with a pair of inverted repeats (IRs) of 26,575 bp. The GC content in the chloroplast genome was 39.23%. In total, 130 genes in the chloroplast genome of *Michelia chartacea* were annotated, including 83 protein-coding genes, 38 tRNA genes, and eight rRNA genes. The phylogenetic analysis showed that *M. chartacea* was closely related with *M. martini* and *M. maudiae*, forming a clade included in *Michelia.*

The Magnoliaceae divided into several smaller genera is a family of lowering plant within the order Magnoliales (Law 1984; Liu 2004; Xia et al. 2009), and is considered as one of the most primitive groups of angiosperms (Li and Guo 2014). *Michelia chartacea* B. L. Chen & S. C. Yang (*Michelia chapensis*) belonging to the magnolia is a dominant tree species in the secondary evergreen broadleaf forests of China, it is even endemic to particular regions where background O_3_ concentrations exceed the thresholds (GB3095-2012) for forest protection in South China (Pan et al. 2020). Analyses of complete chloroplast genomes have the advantage to significantly improve the resolution of phylogenetic relationships in large, complex plant lineages (Doorduin et al. 2011). Due to the characteristics of uniparental inheritance, haploid nature, conserved structure and gene content, small genome size, chloroplast genomes have been widely applied in phylogenetic reconstructions (Dong et al. 2018) and molecular evolution (Walker et al. 2014). Here, the complete cp genome sequence of *M. chartacea* was assembled and analyzed using high-throughput sequencing technology, the annotated cpDNA has been deposited into GenBank with the accession number MT449723.

The fresh leaves were sampled in Longzhong Botanical Garden (32°10′N, 112°10′E), Hubei, China. The specimen of *M. martini* was stored in the herbarium from the Three Gorges University, the accession number was 15005121. The leaves from a single individual *M. chartacea* plant were rapidly frozen with liquid nitrogen and stored at −80 °C until used. Total genomic DNA was extracted using the modifed CTAB method to construct a library for sequencing with Illumina Hiseq 2500 platform (Illumina, San Diego, CA, USA). Additionally, MITObim v 1.8 (https://github.com/chrishah/MITObim) was used to assemble the complete circular cp genome sequence (Hahn et al. 2013). The cp genome was annotated and manually adjusted with CpGAVAS2 (http://www.herbalgenomics.org/cpgavas2) (Shi et al. 2019). The annotated sequence was submitted to NCBI. The genome sequence data that support the findings of this study are openly available in the GenBank of NCBI at https://www.ncbi.nlm.nih.gov/ under the accession no. SUB8488805. The associated BioProject, SRA, and Bio-Sample numbers are PRJNA675703, SRR13045767, and SAMN16711956, respectively.

The chloroplast genome of *M. chartacea* was a closed circular molecule of 160,138 bp presenting a typical quadripartite structure, of which the length of a large single-copy region (LSC) was 88,164 bp and the length of a small single-copy (SSC) region was 18,824 bp, which were separated by the IRA and IRB of 26,575 bp. The contents of CG in the chloroplast genome was 39.23%. A total of 130 chloroplast genes were annotated, containing 83 protein-coding genes (63.85%), 38 transporter RNA genes (29.23%), eight ribosomal RNA genes (6.15%), one pseudogene (0.77%) was inferred to be pseudogenes. Fifteen distinct genes (*trnK-UUU*, *rps16*, *trnS-CGA*, *rpoC1*, *trnL-UAA*, *trnC-ACA*, *petB*, *rp12*, *ndhB*, *trnE-UUC*, *trnA-UGC*, *ycf1*, *ndhA*, *trnA-UGC and trnE-UUC*) contained one intron, while *ycf3* and *clpP* each contained two introns.

To estimate the putative performance of chloroplast genomes on their phylogenetic placements within the family Magnoliaceae, thus, the phylogenetic relationships was performed using the complete cp genomes of *M. chartacea* with those of obtained from 25 other species of Magnoliaceae reported in Genbank of NCBI database based on maximum likelihood (ML) analysis using MEGA 7.0 (Kumar et al. 2016) (https://www.megasoftware.net). The phylogenetic analysis showed that *M. chartacea* was closely related with *M. martini* and *M. maudiae* (Wang et al. 2019), forming a clade included in *Michelia* (Figure 1). The cp genome of *M. chartacea* will provide important data for the further study of Magnoliaceae and systematics of the genus *Michelia*.

**Figure 1.**
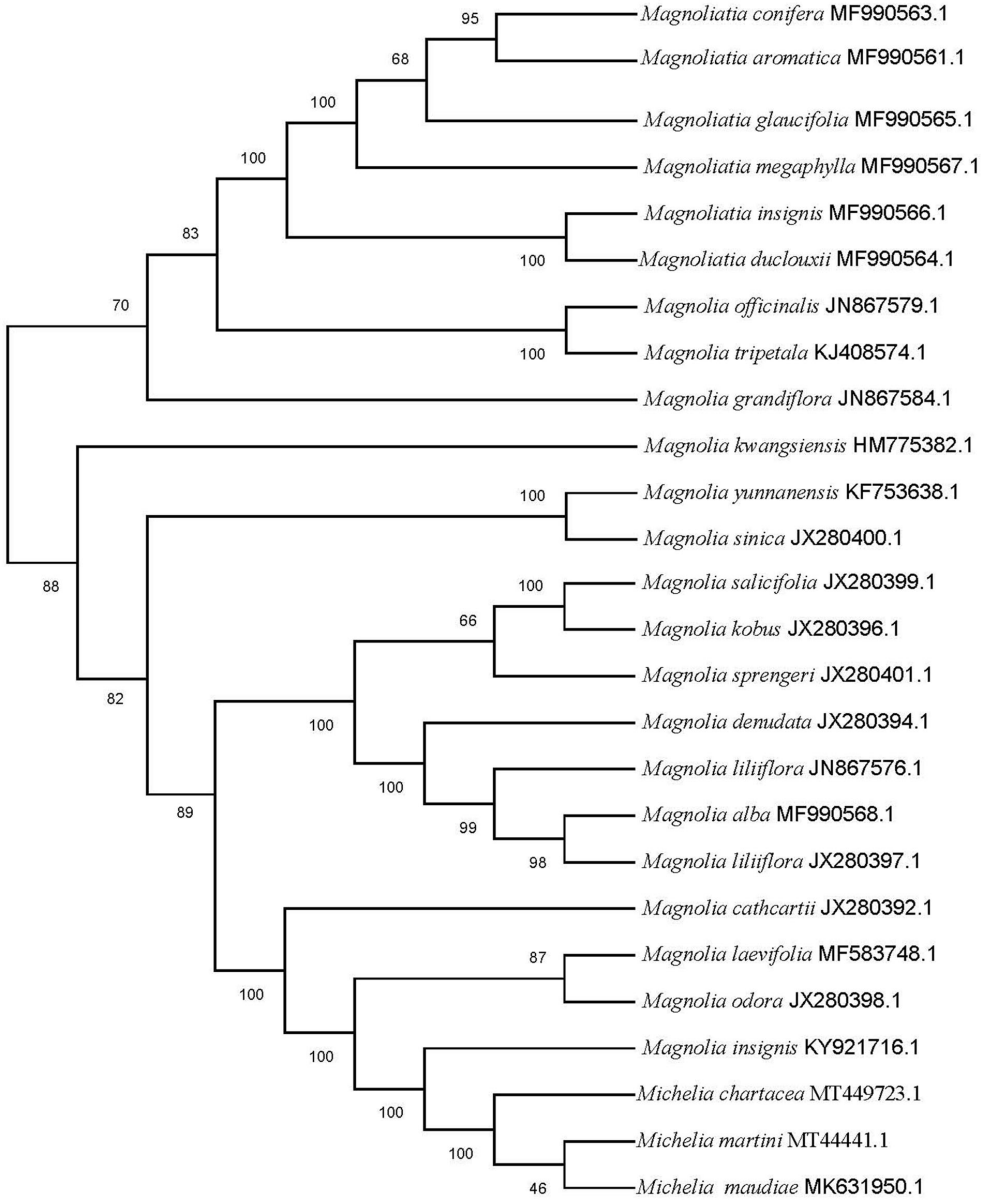
The Maximum likelihood phylogenetic tree of 25 selected Magnoliaceae chloroplast genome sequences. Bootstraps (1000 replicates) are shown at the nodes.

## Data Availability

The data that support the findings of this study are openly available in the National Center for Biotechnology Information (NCBI) at https://www.ncbi.nlm.nih.gov/, reference number MT449723.
